# O038. I.V. methylprednisolone plus diazepam in medication-overuse headache

**DOI:** 10.1186/1129-2377-16-S1-A103

**Published:** 2015-09-28

**Authors:** Matteo Paolucci, Riccardo Altavilla, Giulia Gambale, Claudia Altamura, Fabrizio Vernieri

**Affiliations:** Headache Centre, Policlinico Universitario Campus Bio-Medico di Roma, Rome, Italy

Medication-overuse headache (MOH) is a secondary chronic headache developing as a consequence of prolonged overuse of symptomatic headache drugs for at least 3 consecutive months. It usually, but not invariably, resolves after the overuse is discontinued[1]. Benefit of acute withdrawal of the overused medication has been shown to be effective[2]; performing a transitional therapy (“bridge therapy”) during the days of withdrawal may ensure symptomatic relief from rebound headache and avoid withdrawal symptoms. In this setting, i.v. methylprednisolone may have a protective role[3].

This retrospective study aimed to evaluate the effectiveness of detoxification protocol applied in our Headache Centre, which consists in interruption of the abused drug and in the intravenous administration of methylprednisolone 125 mg plus diazepam 10 mg and esomeprazole 40 mg for 5 consecutive days. Depending on patients’ features, prophylactic therapy for chronic headache was either introduced or modified by the end of the wash-out. We enrolled 36 patients with MOH who underwent wash-out between 2010 and 2014, for whom follow-up data were available. Then we compared these patients with a control group of 21 MOH patients who did not undergo detoxification protocol, but only changed prophylactic therapy. Considered end points were mean monthly days of headache and the percentage of response to treatment at one (T1) and three months (T3): patients were divided into “responders” and “high responders” if reduction was > 50%.

At T0, mean monthly days of headache was 25 in the washout group and 20 in the control group; at T1 means decreased to 8 in the washout group and 11 in the control group (Mann-Whitney U test, p 0.012), with a 68% reduction in the washout group and 40% reduction in the control group. At T3 means were 10 for both groups (p 0.103), with a reduction from baseline of 60% in the washout group and 50% in the control group. At T1 we found 54.4% of “responders” in the washout group, versus 22.8% of responders in the control group (Fisher's exact test, p 0.04); “high responders” rates were 40.4% in the washout group versus 8.8% in the control group (p 0.004). At T3 differences in “responders” and “high responders” rate between the two groups were attenuated, losing statistical significance.

This retrospective study shows that a detoxification protocol with i.v. methylprednisolone and diazepam is widely effective and ensures an adequate reduction of headaches.

Written informed consent to publication was obtained from the patient(s).
